# Recent advances in basic science methodology to evaluate opioid safety profiles and to understand opioid activities

**DOI:** 10.12703/r/10-15

**Published:** 2021-02-19

**Authors:** Aliza T Ehrlich, Emmanuel Darcq

**Affiliations:** 1 Department of Psychiatry and Behavioral Sciences, University of California, San Francisco, CA, USA; 2 Department of Psychiatry, Douglas Research Center, McGill University, Montréal, Canada; 3 INSERM U1114, UNISTRA University of Strasbourg, Strasbourg, France

**Keywords:** GPCR, Network, Signaling, Biased, fMRI, Circuitry, Mu opioid receptor or MOR

## Abstract

Opioids are powerful drugs used by humans for centuries to relieve pain and are still frequently used as pain treatment in current clinical practice. Medicinal opioids primarily target the mu opioid receptor (MOR), and MOR activation produces unmatched pain-alleviating properties, as well as side effects such as strong rewarding effects, and thus abuse potential, and respiratory depression contributing to death during overdose. Therefore, the ultimate goal is to create opioid pain-relievers with reduced respiratory depression and thus fewer chances of lethality. Efforts are also underway to reduce the euphoric effects of opioids and avoid abuse liability. In this review, recent advances in basic science methodology used to understand MOR pharmacology and activities will be summarized. The focus of the review will be to describe current technological advances that enable the study of opioid analgesics from subcellular mechanisms to mesoscale network responses. These advances in understanding MOR physiological responses will help to improve knowledge and future design of opioid analgesics.

## Introduction

The mu opioid receptor (MOR) is the primary molecular target for medicinal opioids^1–4^. MOR activation mediates the unrivalled analgesic properties of opioids, as well as several side effects (constipation, respiratory depression, and abuse liability). Unfortunately, the prescription rate of opioids has increased dramatically in recent years, and a portion of patients with opioid prescriptions develop an opioid use disorder due in part to their strong euphoric effect^5^. Prescribed opioids are now at the center of a rising “opioid epidemic” in North America^6,7^ and in Europe^8,9^, increasing the risk of deaths by overdose. Improving our understanding of how MORs operate at the subcellular level to impact physiology is critical for the development of safer MOR analgesics with more precise painkilling effects.

**Figure 1.**
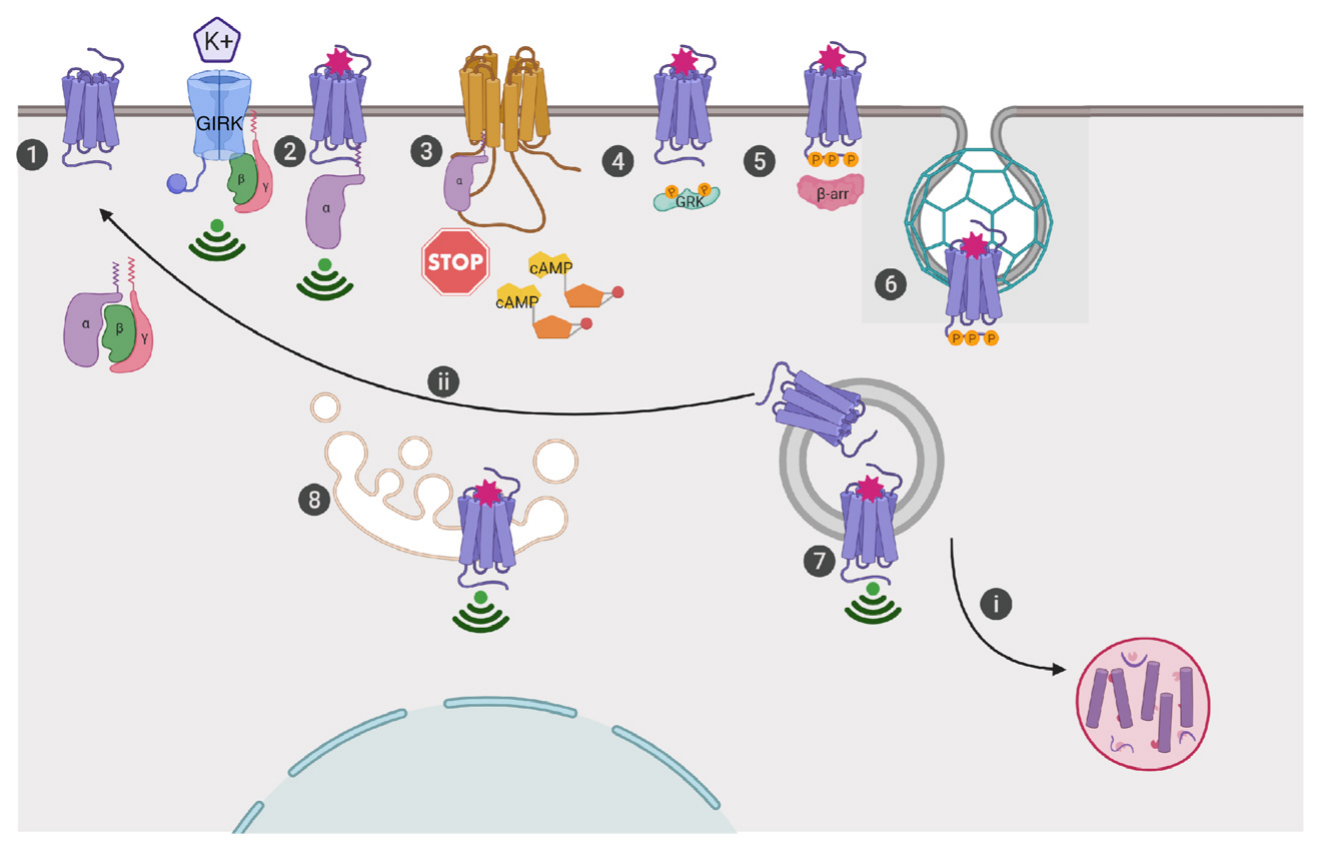
Current view of mu opioid receptor (MOR) trafficking, signaling, and cellular response. **1**. MORs are classically localized to the plasma membrane. **2**. Ligand activation (i.e. morphine binding to the orthosteric site) induces conformational changes in the receptor so that Gαi/o proteins can bind and release Gβγ subunits, which bind to ion channels such as G-protein-coupled inwardly rectifying potassium (GIRK) channels. **3**. Gαi/o proteins inhibit transmembrane adenylyl cyclase-mediated cAMP production. **4**. G-protein-coupled receptor kinases (GRKs) are recruited to phosphorylate MORs. **5**. Adaptor proteins, β-arrestins (β-arr), recognize and bind to the phosphorylated receptor. **6.** MORs are sorted into clathrin-coated pits, which are dynamin dependent. **7**. Endosome vesicles containing MORs pinch off from the membrane, clearing receptors from the cell surface; however, receptors may continue to signal in endosomes, i. be sent to degradation in the lysosome, or ii. be recycled to the membrane for another round of signaling. **8**. Current views also hold that MORs can be activated and signal from internal compartments like the Golgi, if ligands are cell permeable like alkaloids (i.e. morphine)^22,30^.

MORs are G-protein-coupled receptors (GPCRs) that localize to cellular membranes, where they are activated by endogenous peptides or exogenous ligands to induce signaling responses that inhibit neuronal excitability (Figure 1). When activated by agonists, MORs bind to Gαi/o proteins to induce the exchange of GDP for GTP, which in turn releases Gβγ subunits and inhibits adenylyl cyclase from producing cyclic adenosine monophosphate (cAMP). This sequence of events contributes to e.g. altering gene expression^10^, antinociception^11^, and the development of opiate tolerance, dependence, and withdrawal^12^. Opioid receptor activation releases Gβγ subunits, which bind and inhibit L-type voltage-gated calcium channels, which in turn inhibits neurotransmitter release and reduces neurotransmission of pain signals^11^. MOR also induces Gβγ activation of G-protein-coupled inwardly rectifying potassium (GIRK) channels, which allows potassium to flow out of the neuron, resulting in a negative charge or hyperpolarization of the cell and decreased neurotransmission in regions such as ventral tegmental area dopaminergic neurons^13–15^. To remove the receptor from the plasma membrane, GPCR kinases (GRKs) phosphorylate the receptor^16^ and recruited β-arrestin moves the receptor into clathrin-coated pits, where dynamin-dependent endocytosis of the receptors ensues^17,18^. The endosome then transports MORs^19^ to be recycled for another round of activation^20^ or to the degradation pathway via lysosomes^21^.

An essential component for understanding opioid analgesia is improving our fundamental knowledge of the cellular (microscale) and circuit (mesoscale) levels of organization of opioid receptors and their ligands^22^. A main question in the field is whether opioids can produce an analgesic effect without dangerous side effects such as respiratory depression and euphoria. Some evidence already supports this possibility: for example, efforts to produce safer opioids include bypassing the central nervous system altogether. The generation of peripherally limited ligands avoids addictive properties of opioids, such as reward^3^. A pH-sensitive ligand was shown to target peripheral MORs in inflamed tissues, where it produced inflammatory-restricted analgesia without central or intestinal side effects^23,24^. These and other current strategies to design safer opioids have been comprehensively discussed elsewhere^3,25–29^. This review concentrates on describing some of the recent advances in basic science methodology that enable the study of opioid analgesics from microscale to mesoscale network responses. Classical methods employed to study the cellular** mechanisms engaged by the opioid system such as electrophysiology, *in situ* hybridization, and immunohistochemistry are increasingly combined with new techniques (see Table 1) like optogenetic or chemogenetic tools and knock-in (KI) animals and are leading to new insights about the cellular regulation of opioids^31^. Furthermore, new methods in brain imaging are also helping to identify the contribution of MOR activation to physiological effects by mapping the whole activity and synchrony of brain areas. Specifically, magnetic resonance imaging (MRI) is a unique and most informative approach to non-invasively investigate brain anatomy and connectivity of the entire brain in humans and animals. Whole-brain MRI has now been developed in animals to address and follow up, in a longitudinal manner, brain anatomy, functional and structural connectivity patterns, and neurochemistry profiles^32–35^. Some of these approaches may have translational potential to be tested in the clinic and may reveal biological markers, should they correspond to biological parameters that influence or predict the incidence of a disease^36^. Indeed, MRI is an unparalleled non-invasive and versatile method, which makes it feasible to capture the longitudinal effects during different phases of a disease and thus has strong translatability from pre-clinical to human research^37^. Recently, a pharmacological-MRI approach^38,39^ was used to understand whole-brain responses to opioids in living animals^37,40^, suggesting that MRI approaches may soon prove useful for opioid drug development. There are several key methodologies that are actively working to delineate opioid responses at cellular and system levels as well as map and characterize opioid sites of action to improve our understanding of opioids and guide drug development. Selected recent methodologies will be summarized in this review.

**Table 1.** Genetically modified mice available to study the mu opioid receptor (MOR) or MOR-expressing cells discussed in this review.

Mouse line	Mouse line	Findings	Refs
***Detecting MOR expression*** ***and localization in tissues*** ***and in vivo***			
Knock-in *MOR* reporter mice	*MOR* with a C-terminal mCherry fusion	MOR expression was visualized at the brain, neuron, and subcellular level with an enriched expression in medial habenula	54,55
	*MOR* with a C-terminal Venus fusion	Different MOR internalization profiles were visualized depending on MOR agonists	45
***Manipulating MOR-*** ***expressing neurons***			
Knock-in *MOR-Cre* mice	*MOR-Cre mouse with a T2A cleavable peptide-* *Cre recombinase*	Fine mapping of MOR striatal projection neurons in the patch compartment of the striatum	56
	*MOR-Cre mouse with a T2A cleavable peptide* *and tamoxifen-inducible Cre recombinase*	n/a	57
	*MOR-Cre with a T2A cleavable peptide and* *Cre recombinase fused to an enhanced green* *fluorescent protein*	Activation of ventral tegmental area-MOR neurons produced a strong place aversion	58

## Investigating ligand-directed functional selectivity at MORs

At the microscale, opioid responses begin with ligand binding the receptor to initiate signaling cascades. There are several well-characterized pathways for receptor regulation. Early observations of agonist selective regulation of MOR activity^19,41,42^ motivated the idea now known as “functional selectivity” or “biased agonism”, suggesting that two distinct agonists which activate the same receptor may produce different signaling responses and perhaps physiological outcomes^43,44^. A classic example of ligand-directed selectivity at the MOR is the agonist morphine, which, at the molecular level, fully activates G-protein signaling but weakly internalizes receptors *in vivo* (in animal experiments)^42^ and *in vitro*^45^. In contrast, the potent and non-selective opioid agonist etorphine, at the molecular level, internalizes receptors robustly and rapidly in animal experiments and *in vitro*^41,46^. These early observations of different receptor internalization responses to two opioid agonists suggested distinct agonist-directed regulation of MORs, which may explain differences in observed physiological responses of these two agonists. Besides G-protein regulation, MORs are also regulated by phosphorylation through kinase activity like GRKs, JNKs, and PKCs^47–49^ and through the recruitment of β-arrestins^50^, which can also drive additional receptor-mediated signaling events at internal membrane compartments^51,52^.

**Figure 2.**
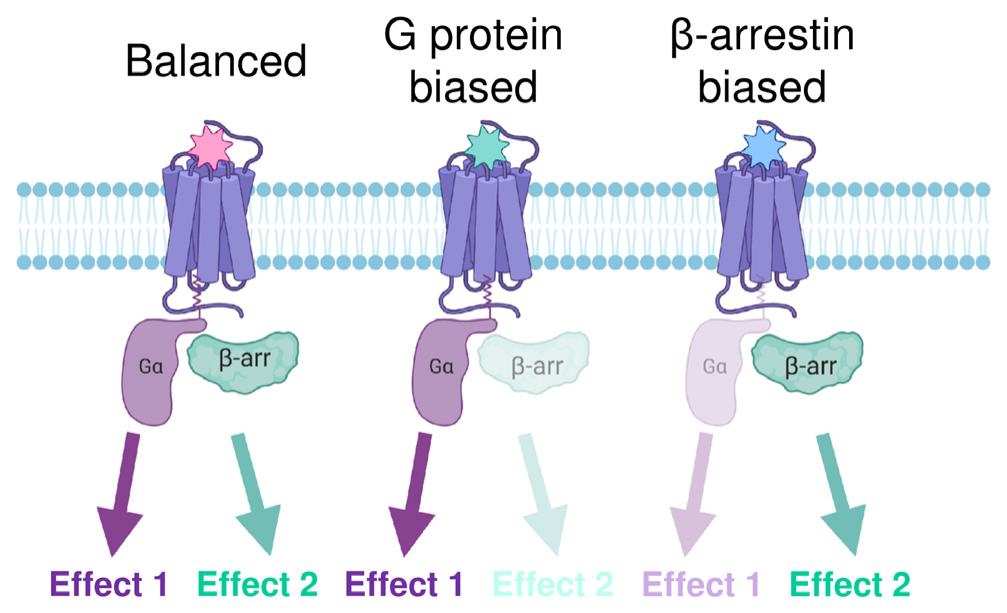
Biased agonist-induced signaling. Agonists that efficiently induce opioid receptor activation of both G-protein and β-arrestin are thought of as balanced or unbiased ligands. If either G-protein or β-arrestin effects are more efficiently activated by a ligand, they are known as biased agonists^68^.

Following the discovery that β-arrestin knockout (KO) animals treated with morphine showed enhanced antinociception with decreased respiratory depression and constipation^53^, the “arrestin hypothesis” or the idea that β-arrestin mediates adverse side effects of MOR activation further motivated the academic field and industry to design G-protein-biased MOR agonists or drugs that would activate β-arrestin less than the G-protein pathway (Figure 2)^27^. The novel agonists that have emerged since, including TRV-130, commercially named OLINVYK®^59^, PZM21^60^, and SR17018^61^, were proposed to be potent G-protein activators with high analgesic effect, low β-arrestin recruitment, and low respiratory depression and thus greater therapeutic windows.

Since these new G-protein-biased ligands have been made available to the scientific community, additional studies have revealed results that should also be considered when designing G-protein-biased MOR drugs. For example, in one report, PZM21 has been shown to produce respiratory depression to a similar extent as morphine^62^; however, in another study, minimal respiratory depression was observed with much higher concentrations of PZM21 in mice^63^, and the adverse condition of hyperalgesia has been observed at low non-antinociceptive doses of PZM21 in rats^64^. TRV-130 was found to retain side effects, including constipation in mice^65^ and reward in rats^66^. Finally, in agreement with the original paper, SR17018^61^ has been shown to produce minimal respiratory depression in comparison to other MOR agonists, albeit with a limited dosage window because of the use of a less-soluble salt version than the original SR17018 and using different methods to measure and quantify respiratory depression in mice^63^. Additionally, while bias toward the G-protein pathway has been the sought-after hallmark of a safer MOR ligand, the contribution of the β-arrestin pathway to respiratory depression remains unclear^67,68^. Morphine still induces respiratory depression in mice which have MORs that are phosphorylation deficient and thus do not recruit β-arrestins^69^. Moreover, a recent study shows that β-arrestin KO mice do exhibit morphine-induced respiratory depression and constipation^70^, challenging the role of the “arrestin hypothesis” in MOR adverse effects. Nevertheless, TRV-130 (OLINVYK®) has gone on to clinical trials in Asia and Europe and has recently been approved by the FDA in the U.S. as an intravenous drug for the management of moderate-to-severe acute pain^71,72^. In clinical trials, OLINVYK® provided potent analgesia that was superior to that observed in patients treated with placebo and was accompanied by a lower occurrence of adverse events, such as respiratory and gastrointestinal events, compared to morphine^72,73^. These findings suggest that while the contribution of β-arrestin signaling to respiratory depression remains a subject of investigation, G-protein-biased agonists which exhibit lower respiratory depression than balanced opioid drugs do hold promise for safer pain management with opioids.

In a recent paper, the concept of biased agonism was challenged as the reason for the observed improved safety profiles of PZM21, TRV-130, and SR17018. The authors^63^ suggest an alternative mechanism of how low intrinsic efficacy of these compounds can distort the interpretation of highly amplified G-protein activity assays and can account for confounding factors that impact the operational model^74^ used to calculate bias between G-protein and arrestin pathways. Indeed, overexpressed receptor expression systems have shown that MOR agonist efficacy in signaling varies widely across drugs but correlates with receptor internalization^75^. The authors show that in transiently transfected cells, comparable assessment of compounds across signaling pathways accounting for variation in kinetics, temporal location of receptors, cell systems, receptor reserve, amplified detection methods, and inclusion of therapeutic window calculation in mice find PZM21, TRV-130, and SR17018 to be low intrinsic efficacy compounds rather than G-protein biased^63^. Importantly, depending on the analysis methodology, these data may also support the fact that PZM21, TRV-130, and SR17018 are G-protein-biased agonists, as suggested in a recent re-analysis of Gillis *et al*.’s work^76^. These studies demonstrate how biased agonism at MORs is still an emerging area and that MOR signaling activities are a hotly discussed area of investigation.

Intriguingly, G-protein bias has also been observed with endogenous opioid ligands. Most recently, functional selectivity was examined by GTPγS and β-arrestin recruitment for 22 endogenous opioid peptides^77^. In addition to measuring bias, the authors demonstrated that binding activity of classically distinct peptides (enkephalins, endorphins, and dynorphins) was shown at all three opioid receptors, thus suggesting that, evolutionarily, opioid receptors are designed to bind multiple ligands that direct distinct signaling and physiological outcomes. Furthermore, it is important to assess drug activities at native MORs in neurons. Recently, MOR agonist-directed receptor redistribution in native neurons was shown to correlate well with overexpressed receptor internalization in HEK-293 cells, suggesting that MOR agonist activities can be evaluated in native receptor conditions by measuring receptor trafficking^45^.

Remarkably, in three completely independent studies, a partial MOR agonist, buprenorphine, performs like the ultimate opioid, with rapid and potent G-protein activity, negligible arrestin recruitment or receptor internalization^45,63,75^, rapid and sustained antinociception in hotplate test, and low respiratory depression effects^63^. Buprenorphine thus appears to be effective in experimental nociceptive assays and is a current mainstay treatment for opioid use disorder^78^. Some caveats exist, though; buprenorphine may be difficult to prescribe for pain owing to its restricted regulation as a schedule III drug in the U.S., complex pharmacology including off-target effects at other opioid receptors such as antagonism at Kappa, and its delicate dosing and limited bioavailability^79–81^. Collectively, the ideal opioid would have a wide therapeutic window and be a potent analgesic, with limited euphoria and respiratory depression. Thus far, opioids such as buprenorphine, PZM21, TRV-130, and SR17018 at the system level are antinociceptive and have lower respiratory depression in animal studies; at the molecular level, they also have in common miniscule loss of receptor from the cell surface, weak GRK recruitment, weak arrestin recruitment, weak receptor translocation to endosomes, and similar phosphorylation barcodes detected by western blotting. Designing new opioids which satisfy all these criteria will hopefully yield good candidates for clinical trials soon.

## Visualizing opioid receptors *in vivo* using KI animals

In the last 15 years, since the creation of fluorescent opioid receptor KI mice^82^, MOR-KI mice have become a valuable tool in deciphering the role of these receptors at the circuit and cellular level^54,83–85^. Here, we focus on the MOR and how knowledge acquired with MOR-KI may serve to improve our understanding of opioid analgesia. On the anatomical level, mapping of MORs using MOR-mCherry mice^55^ has revealed prominent receptor populations in the diencephalic conduction system, including the striatum, septum, habenula, and interpeduncular nucleus^54^. The particularly unique pattern of MOR in the medial habenula, a dense brain area important for aversion and inhibitory control^86–88^, has raised questions about the role of MORs in the medial habenula^89^ and given insight into opioid analgesia circuitry^90^. Additionally, extensive mapping of MOR-mCherry mice crossed with DOR-eGFP mice^55^ revealed the existence of colocalized and coimmunoprecipitated complexes of MOR and DOR receptors, called receptor heteromer populations. The DOR-eGFP/MOR-mCherry animals have become a critical tool (for review, see 91) for investigations of these potential therapeutic targets, MOR-DOR heteromers, in antinociception *in vitro*^92^ and *in vivo*^93^. Furthermore, MOR-mCherry mice alone have been used to dispute and discern glial cell populations, such as the lack of MOR expression in spinal microglia^93^ or presence of MOR in astrocytes of the hippocampus^84^. More recently, MOR-Venus mice were created employing the highly photo- and thermo-stable fluorescent protein Venus, adding resonance energy transfer capabilities^45^. Mapping of MOR-Venus signals in whole mouse brain revealed a MOR expression profile in agreement with MOR-mCherry anatomy^55^, showing how robust the fluorescent receptor KI mouse approach is 45. MOR-Venus mice were used to identify agonist-directed receptor trafficking profiles for biased and classic MOR agonists in neurons, which correlated to receptor activities in overexpression systems, supporting the relevancy of biased MOR agonists to physiology^45^. Additionally, KI mice can be used to probe *in vivo* protein–protein interactions, as the fluorescent fusion proteins or epitope tags are amenable to proteomic approaches. This has recently been shown for the N-terminally tagged delta opioid receptor^94^, which if extended to MORs would help improve our understanding of the proteins interacting directly with MOR, called the MOR interactome. Collectively, these KI genetic approaches allow basic scientists to trace endogenous MOR activities using fluorescence and immunoreactive methods. Caveats to this method include the possibility that altering the endogenous protein by addition of a short sequence in the case of an epitope tag or by addition of a fluorescent protein could impact the behavior of the receptor and its interactions with proteins and ligands. However, to date, this is the best tool that we have to directly observe endogenous receptor activities at the molecular level. Thus, fluorescent receptor KI animals are excellent tools to investigate novel opioid effects at endogenous receptors on cellular and whole animal levels.

## Distinguishing agonist effects by probing MOR conformational states

Direct observation of drug-induced receptor activation in cells has not been possible until recently. New advances in structural biology have led to the generation of such probes that can be employed to distinguish receptor conformational states in transfected cell culture systems. Two types of probes derived from structural studies that are being used to study opioid receptor proximal activities are nanobodies, also known as single-domain antibodies^95,96^, and mini-G proteins, which are composed of the Ras-like domain of G-protein α subunits^97,98^. Nb33, a nanobody that binds selectively to a specific antigen, was first demonstrated to be a MOR and delta receptor proximal conformational biosensor in an elegant study discerning peptide from non-peptide opioid agonist-induced receptor activities in cultured cells (MOR transfected HEK-293 and rat embryonic striatal neurons)^99^.

Additionally, this opioid biosensor offers the ability to study receptor proximal events, which can help to distinguish direct ligand-induced receptor activities from indirect downstream signaling^100^ or examine agonist-induced intrinsic efficacy^63^. In recent research using these tools, the authors demonstrated how Nb33 and mini-G fluorescent engineered probes can detect distinct opioid receptor states induced by ligands coupling to the receptor^100^. For example, in a TIRF microscopy assay in HEK-293 cells, etorphine recruited both mini-Gsi and Nb33 equally, whereas DAMGO, morphine, and PZM21 had higher potency for mini-Gsi, with the latter two having higher efficacy for mini-Gsi over Nb33. Intriguingly, the semi-synthetic natural product mitragynine pseudoindoxyl^101^ recruited mini-Gsi but not Nb33, supporting the idea that, in controlled cell culture studies, distinct ligands induce selective conformational changes of receptors that may impact receptor signaling and regulation. This is an active area of investigation, and the implications of ligand-induced distinct conformational changes on MOR activities have yet to be directly assessed *in vivo* and ultimately in controlled clinical trials.

Enabling the study of endogenous MORs has also been helped by recent advances in traceless affinity labeling that have now made it possible for ligand-directed irreversible fluorescent tagging of endogenous MOR in rat and mouse brain slices^102^ that will allow for the tracing of endogenous MOR activities *in vivo* in response to biased agonists and could be applied to primary cultured neuron studies. For now, these studies are mainly done in cultured cells or slices, and in the future it will be important to determine if these MOR activities are also seen in post-mortem or immunopluripotent stem cell-derived human neurons.

## Cellular contributions of time and space to MOR signaling

MOR signaling from the plasma membrane has been the prevailing view as the site of its action. However, a growing number of GPCRs have been shown to signal from internal compartments (Figure 1)^103–106^. Recently, remarkable tools have revealed that opioid receptors can signal from subcellular compartments, including endosomes and Golgi. Employing advanced microscopy techniques, Stoeber and co-authors showed that in mammalian cells and striatal neurons, peptide opioids differ from non-peptide opioids in that the former activate receptors first at the plasma membrane and then at endosomes whereas the latter activate opioid receptors at a third location, Golgi outposts^99^. These findings may help to explain distinct opioid-induced cellular effects, in particular, by identifying how synthetic opioid drugs like morphine, which signals at Golgi, contribute to distinct signaling responses compared to endogenous opioids. In the clinic, peptides are not prescribed owing to their low activity after systemic administration. However, their potential for drug development has been reviewed elsewhere^107–109^, and these studies highlight the need to take the location of signaling receptors into account when designing new drugs.

MORs are also found on axons, and the contribution of presynaptic MORs to MOR cellular biology has not been well understood. Recent work in rat primary neurons using super resolution microscopy and MORs fused with a fluorescent reporter emitting fluorescence in function of the acidity of the environment (pH-sensitive GFP-tagged MORs) demonstrated that MORs are laterally mobile on the axonal surface and locally recycle separately from the synaptic vesicle cycle, which may have considerable impact on understanding MOR agonist-induced presynaptic inhibition and neuromodulation^110^. How distinct opioid nonpeptide or peptide agonists impact MORs in axons and various neuronal subcellular locations remains to be seen. Perhaps these questions will be addressed using emerging new tools and techniques. For example, a recent proteomic approach known as APEX combines proximity-based biotinylation with mass spectrometry to allow for quantitative, time-resolved measurement of GPCR agonist response in living cells^111^. When combined with spatial references, proteins that delineate precise cellular compartments, this technique allows for the detection of GPCR–protein interaction networks resolved in time and space^112^. If adapted to living neurons, this approach could help to distinguish local signaling molecular events at presynaptic receptors from postsynaptic receptors and potentially identify more precise therapeutic targets. Remarkably, a recent study on the delta opioid receptor demonstrates that activating endosome-localized delta receptors of nociceptors using innovative nanoparticle-encapsulated agonists cause a long-lasting inhibition of neuronal excitability^113^, suggesting that spatiotemporal organization of receptor activities impacts opioid physiology. These studies and others demonstrate that spatial location of opioid receptor signaling is a rapidly developing area of investigation and an important factor in understanding opioid mechanisms of action which could yield new therapeutics that target more precise receptor populations.

## Manipulating MOR-expressing neurons to identify function(s) of opioid circuitry using KI animals

Combining Cre technology (genetic modification using Cre recombinase in animals) with chemogenetics (genetically modified proteins engineered to be activated with a small molecule) or optogenetics (genetic manipulation of specific neuronal populations by using light to control neurons expressing light-sensitive ion channels) allows for the mapping or manipulation of select neurons or to even record neuronal activity using fluorescent calcium indicators (genetically engineered calcium biosensors) in Cre-positive neurons^114–117^. New genetic tools were recently designed to manipulate neurons expressing opioid peptides or opioid receptors^2,118^. Similar tools have already been used to determine distinct novel functions of neurons expressing opioid peptides. For example, it was demonstrated that preprodynorphin (Pdyn)-expressing neurons in the nucleus accumbens shell may drive opposite behavior (aversion or preference) depending on the localization of these neurons in the shell subregion (ventral versus dorsal)^119^. Furthermore, animals that express a Cre-recombinase under proopiomelanocortin or proenkephalin promoters were generated and anatomically characterized^120^ and will be interesting to use in pain- or addiction-related studies to determine the adaptations occurring in opioid circuitries. Tools to target opioid receptor neurons are also now available. KOR-Cre was the first KI mouse generated and characterized, in which opioid receptor-positive neurons express Cre-recombinase^121^.

Recently, three independent laboratories designed, generated, and characterized in parallel three novel genetic tools, allowing the targeting and manipulation of specific neurons expressing MOR *in vivo*. Märtin *et al*. generated a MOR-Cre mouse strain by inserting a Cre recombinase with a peptide that is autocleavable, allowing the separation of the MOR and Cre proteins, called T2A cleavable peptide-Cre recombinase, that was inserted into the fifth exon of the gene coding for MOR (*Oprm1*)^56^. This MOR-Cre mouse strain was crucial to map MOR striatal projection neurons in the patch compartment of the striatum^56^. Okunomiya *et al*. designed and characterized an inducible MOR-Cre KI mouse strain, in which the stop codon, which signals the termination of the translation process of the *Oprm1* gene, was replaced by a DNA fragment encoding a T2A cleavable peptide and an inducible Cre recombinase^57^. The inducible Cre recombinase is made by the fusion of a mutated ligand-binding domain of the estrogen receptor to the Cre recombinase variant, which made this inducible Cre activated only after delivery of tamoxifen. Finally, Bailly *et al*. generated a MOR-Cre mouse strain by inserting a T2A cleavable peptide and Cre recombinase fused to a fluorescent reporter protein (enhanced GFP), allowing the detection of the Cre and thus the MOR neurons, in the fourth exon of the *Oprm1* gene^58^. This mouse strain was characterized, and MOR signaling was intact, as demonstrated by typical morphine-induced antinociception, sensitization, and the classic morphine-induced hyperlocomotion in mice^58^. Bailly and co-authors used the MOR-Cre animals to activate ventral tegmental area-MOR neurons by optogenetics, and, as it was anticipated from literature that has shown how opioids inhibit these neurons and produce reward, the opposite action by optogenetic activation of these neurons produced a strong aversion^58^. These mouse lines will be critical for future investigation into the functions of opioid-responsive neurons in antinociception and side effects such as respiratory depression to determine how subsets of MOR neurons can operate in precise circuits to contribute to opioid physiology and adaptations. For example, MOR-Cre mice will help to delineate the role of MOR neurons of the pre-Bötzinger complex, as it was shown recently, using local deletion of the MOR in mice, that opioid activation of MOR neurons of the pre-Bötzinger complex seems to have the strongest impact on respiratory depression^122^.** Another application of these MOR-Cre lines may be to study the activity of MOR-expressing neurons using calcium imaging. For example, time-lapse *in vivo* calcium imaging was used to identify a neural ensemble in the basolateral amygdala that mediates unpleasantness in an animal model of persistent nociceptive stimulation^123^, and targeting these neurons may reduce nociceptive responses without increasing reward. Furthermore, similar studies using MOR-Cre mice may help to identify opioid-responsive circuitry implicated in unpleasantness associated with nociceptive stimulation. Finally, it will be crucial in the future to engineer safe viral-mediated therapeutics to target specific neuronal populations in humans.

The creation of these MOR-Cre strains will allow the scientific community to map and chemogenetically or optogenetically manipulate or record neuronal activity of opioid-responsive neurons to improve understanding of opioid-responsive neuronal networks and their adaptations and may thus identify new concepts relevant for opioid analgesia. Altogether, these new Cre lines are opening doors for both mapping and functional studies of opioid receptor-/peptide-expressing neurons and may be crucial to understand opioid analgesia.

## Effects of analgesic opioids on whole-brain connectivity and activity using MRI

Different types of environmental stimuli, such as pain or repeated opioid use, cause neuroadaptations. These adaptations can impact whole-brain function. A method to evaluate brain connectivity and activities at the whole scale level with translational potential is MRI. The complexity of human brain networks starts to be transposable in rodents, as similar resting state networks are found in rodents and humans^124^, which allows for parallel non-invasive longitudinal experiments in living humans and animals. Recently, pharmacological MRI has emerged as a method to map brain activation following acute effects of opioid analgesics in humans and rodents. Pharmacological MRI is a highly powerful method to understand brain responses to drugs^38,39^ and has been developed in rodents and humans^125–128^. In opioid-naive individuals, a morphine or buprenorphine challenge altered blood-oxygen-level-dependent (BOLD) signals in the periaqueductal grey^37,129^. Additionally, in humans, oxycodone reduced and disrupted functional connectivity between anterior cingulate cortex and insula or dorsal striatum centers belonging to the pain and reward centers^130^. Nevertheless, the overall impact of opioid exposure on brain network activities remains poorly understood.

The MOR-mediated adaptations contributing to these whole-brain effects have started to be investigated, and animal MRI may be the most appropriate tool to reach this goal, as animals with MOR (wild-type [WT]) or without (KO) are available^1^. First, it was demonstrated that the deletion of MOR is enough to alter resting-state functional connectivity^131^. Interestingly, the strongest modification occurred in the reward/aversion circuitry, including regions important for analgesia, such as the periaqueductal gray. Secondly, pharmacological MRI analysis enabled the determination of oxycodone patterns of brain activation^132^ and oxycodone effects on whole-brain functional connectivity^40^. In the latter study, oxycodone effect was assessed in WT and MOR-KO animals to extract MOR-dependent whole-brain functional connectivity signatures using functional MRI (fMRI). Importantly, almost no effect of oxycodone was found in MOR-KO mice, indicating that the oxycodone whole-brain functional connectivity signatures are MOR mediated^40^. Using a data-driven analysis (independent component analysis), it was demonstrated that oxycodone produced a reduction of functional connectivity across 71 components including the isocortex, nucleus accumbens, and periaqueductal gray, brain regions important for the antinociceptive effects of opioids^40^. Furthermore, focused and hypothesis-oriented analysis (seed-to-seed) presented highest functional connectivity reduction between the periaqueductal gray and nucleus accumbens, showing that oxycodone impacts brain activity in reward and aversion/pain networks^40^.

Since rodent fMRI is still emerging^133^, more effort should be put into applying resting-state fMRI to understand opioid analgesia. fMRI studies on the opioid system will be necessary to study the effect of opioid circuitry on whole-brain communication and to determine how distinct opioids act on reward, respiratory depression, or pain networks, and if distinct opioids are acting differently on these networks. The hope will be to find an opioid analgesic targeting only the pain networks without affecting centers such as hubs implicated in reward or respiration. This may be achieved using fMRI in rodents by first identifying the brain networks activated by classical opioids like morphine and then comparing to novel opioid drugs that produce limited unwanted effects on reward and respiration. A comparison of opioid drugs may reveal a signature with which future opioid drug design would aim to reach. Furthermore, rodent MRI combined with optogenetic^134,135^ or chemogenetic^136,137^ tools can identify the impact of neuronal populations on whole-brain activity. These methods may eventually allow for the discovery of the link between microcircuit opioid activity and the brain network at the mesoscale level. For example, chemogenetic manipulations and fMRI were combined recently to map serotonergic transmission, and the stimulation of serotonergic activity induced particular activation of brain regions covering cortico-hippocampal and ventro-striatal areas important for depressive-like behaviors^138^. Further studies, to determine the effect of local manipulation of MOR circuitry on whole-brain communication using MOR-Cre animals, may help to characterize opioid-responsive circuitries with the goal to improve opioid analgesics. Altogether, these recent studies demonstrate that high-resolution fMRI is possible in rodents combined with diverse genetic tools and holds potential for translation as similar longitudinal studies will be possible in humans and rodents.

## Conclusion

Altogether, recent advances in the characterization of MORs will help to determine future strategies to develop a MOR agonist that alleviates pain and has fewer side effects. The new methodologies discussed here represent a selection of studies employing exciting new tools being developed to study MORs, all the way from subcellular mechanisms to mesoscale network responses. These studies will enhance our knowledge on opioid analgesia and physiology, guide future research, and innovate opioid pharmacology.

## References

[1] Matthes HW Maldonado R Simonin F: Loss of morphine-induced analgesia, reward effect and withdrawal symptoms in mice lacking the mu-opioid-receptor gene. *Nature.* 1996; 383(6603): 819–23. 10.1038/383819a0 8893006

[2] Darcq E Kieffer BL: Opioid receptors: Drivers to addiction? *Nat Rev Neurosci.* 2018; 19(8): 499–514. 10.1038/s41583-018-0028-x 29934561

[3] Stein C: New concepts in opioid analgesia. *Expert Opin Investig Drugs.* 2018; 27(10): 765–75. 10.1080/13543784.2018.1516204 30148648

[4] Valentino RJ Volkow ND: Untangling the complexity of opioid receptor function. *Neuropsychopharmacology.* 2018; 43(13): 2514–20. 10.1038/s41386-018-0225-3 30250308 PMC6224460

[5] Vowles KE McEntee ML Julnes PS: Rates of opioid misuse, abuse, and addiction in chronic pain: A systematic review and data synthesis. *Pain.* 2015; 156(4): 569–76. 10.1097/01.j.pain.0000460357.01998.f1 25785523

[6] Kolodny A Courtwright DT Hwang CS: The prescription opioid and heroin crisis: A public health approach to an epidemic of addiction. *Annu Rev Public Health.* 2015; 36: 559–74. 10.1146/annurev-publhealth-031914-122957 25581144

[7] Compton WM Jones CM Baldwin GT: Relationship between Nonmedical Prescription-Opioid Use and Heroin Use. *N Engl J Med.* 2016; 374(2): 154–63. 10.1056/NEJMra1508490 26760086 PMC11784537

[8] Chenaf C Kaboré JL Delorme J: Prescription opioid analgesic use in France: Trends and impact on morbidity-mortality. *Eur J Pain.* 2019; 23(1): 124–34. 10.1002/ejp.1291 30051548

[9] Jannetto PJ Helander A Garg U: The Fentanyl Epidemic and Evolution of Fentanyl Analogs in the United States and the European Union. *Clin Chem.* 2019; 65(2): 242–53. 10.1373/clinchem.2017.281626 30305277

[10] McClung CA Nestler EJ Zachariou V: Regulation of gene expression by chronic morphine and morphine withdrawal in the locus ceruleus and ventral tegmental area. *J Neurosci.* 2005; 25(25): 6005–15. 10.1523/JNEUROSCI.0062-05.2005 15976090 PMC6724795

[11] Corder G Castro DC Bruchas MR: Endogenous and Exogenous Opioids in Pain. *Annu Rev Neurosci.* 2018; 41: 453–73. 10.1146/annurev-neuro-080317-061522 29852083 PMC6428583

[12] Nestler EJ Alreja M Aghajanian GK: Molecular and cellular mechanisms of opiate action: Studies in the rat locus coeruleus. *Brain Res Bull.* 1994; 35(5–6): 521–8. 10.1016/0361-9230(94)90166-x 7859110

[13] Harris GC Williams JT: Transient homologous mu-opioid receptor desensitization in rat locus coeruleus neurons. *J Neurosci.* 1991; 11(8): 2574–81. 10.1523/JNEUROSCI.11-08-02574.1991 1651377 PMC6575524

[14] Lüscher C Slesinger PA: Emerging roles for G protein-gated inwardly rectifying potassium (GIRK) channels in health and disease. *Nat Rev Neurosci.* 2010; 11(5): 301–15. 10.1038/nrn2834 20389305 PMC3052907

[15] Johnson SW North RA: Two types of neurone in the rat ventral tegmental area and their synaptic inputs. *J Physiol.* 1992; 450: 455–68. 10.1113/jphysiol.1992.sp019136 1331427 PMC1176131

[16] Komolov KE Benovic JL: G protein-coupled receptor kinases: Past, present and future. *Cell Signal.* 2018; 41: 17–24. 10.1016/j.cellsig.2017.07.004 28711719 PMC5722692

[17] Shenoy SK Lefkowitz RJ: β-Arrestin-mediated receptor trafficking and signal transduction. *Trends Pharmacol Sci.* 2011; 32(9): 521–33. 10.1016/j.tips.2011.05.002 21680031 PMC3159699

[18] Oakley RH Laporte SA Holt JA: Differential affinities of visual arrestin, beta arrestin1, and beta arrestin2 for G protein-coupled receptors delineate two major classes of receptors. *J Biol Chem.* 2000; 275(22): 17201–10. 10.1074/jbc.M910348199 10748214

[19] Keith DE Murray SR Zaki PA: Morphine activates opioid receptors without causing their rapid internalization. *J Biol Chem.* 1996; 271(32): 19021–4. 10.1074/jbc.271.32.19021 8702570

[20] Tsao P Cao T von Zastrow M: Role of endocytosis in mediating downregulation of G-protein-coupled receptors. *Trends Pharmacol Sci.* 2001; 22(2): 91–6. 10.1016/s0165-6147(00)01620-5 11166853

[21] Tsao P von Zastrow M: Downregulation of G protein-coupled receptors. *Curr Opin Neurobiol.* 2000; 10(3): 365–9. 10.1016/s0959-4388(00)00096-9 10851176

[22] Jullié D Gondin AB von Zastrow M: Opioid Pharmacology under the Microscope. *Mol Pharmacol.* 2020; 98(4): 425–32. 10.1124/mol.119.119321 32198210 PMC7562971

[23] Spahn V Del Vecchio G Labuz D: A nontoxic pain killer designed by modeling of pathological receptor conformations. *Science.* 2017; 355(6328): 966–9. 10.1126/science.aai8636 28254944

[24] Spahn V Del Vecchio G Rodriguez-Gaztelumendi A: Opioid receptor signaling, analgesic and side effects induced by a computationally designed pH-dependent agonist. *Sci Rep.* 2018; 8(1): 8965. 10.1038/s41598-018-27313-4 29895890 PMC5997768

[25] Ehrlich AT Kieffer BL Darcq E: Current strategies toward safer mu opioid receptor drugs for pain management. *Expert Opin Ther Targets.* 2019; 23(4): 315–26. 10.1080/14728222.2019.1586882 30802415 PMC6497449

[26] Pradhan AA Smith ML Kieffer BL: Ligand-directed signalling within the opioid receptor family. *Br J Pharmacol.* 2012; 167(5): 960–9. 10.1111/j.1476-5381.2012.02075.x 22708627 PMC3492979

[27] Madariaga-Mazón A Marmolejo-Valencia AF Li Y: Mu-Opioid receptor biased ligands: A safer and painless discovery of analgesics? *Drug Discov Today.* 2017; 22(11): 1719–29. 10.1016/j.drudis.2017.07.002 28743488 PMC6620030

[28] Siuda ER Carr R 3rd Rominger DH: Biased mu-opioid receptor ligands: A promising new generation of pain therapeutics. *Curr Opin Pharmacol.* 2017; 32: 77–84. 10.1016/j.coph.2016.11.007 27936408

[29] Kieffer BL: Drug discovery: Designing the ideal opioid. *Nature.* 2016; 537(7619): 170–1. 10.1038/nature19424 27533037

[30] Williams JT Ingram SL Henderson G: Regulation of μ-opioid receptors: Desensitization, phosphorylation, internalization, and tolerance. *Pharmacol Rev.* 2013; 65(1): 223–54. 10.1124/pr.112.005942 23321159 PMC3565916

[31] Valentino RJ Koroshetz W Volkow ND: Translating Opioid Pharmacology From Bench to Bedside, and Back. *Biol Psychiatry.* 2020; 87(1): 4–5. 10.1016/j.biopsych.2019.05.026 31806084

[32] Silverman JL Ellegood J: Behavioral and neuroanatomical approaches in models of neurodevelopmental disorders: Opportunities for translation. *Curr Opin Neurol.* 2018; 31(2): 126–33. 10.1097/WCO.0000000000000537 29493556 PMC5846342

[33] Dopfel D Zhang N: Mapping stress networks using functional magnetic resonance imaging in awake animals. *Neurobiol Stress.* 2018; 9: 251–63. 10.1016/j.ynstr.2018.06.002 30450389 PMC6234259

[34] Choi JK Dedeoglu A Jenkins BG: Application of MRS to mouse models of neurodegenerative illness. *NMR Biomed.* 2007; 20(3): 216–37. 10.1002/nbm.1145 17451183

[35] Gozzi A Schwarz AJ: Large-scale functional connectivity networks in the rodent brain. *NeuroImage.* 2016; 127: 496–509. 10.1016/j.neuroimage.2015.12.017 26706448

[36] Strimbu K Tavel JA: What are biomarkers? *Curr Opin HIV AIDS.* 2010; 5(6): 463–6. 10.1097/COH.0b013e32833ed177 20978388 PMC3078627

[37] Becerra L Upadhyay J Chang PC: Parallel buprenorphine phMRI responses in conscious rodents and healthy human subjects. *J Pharmacol Exp Ther.* 2013; 345(1): 41–51. 10.1124/jpet.112.201145 23370795

[38] Jonckers E Shah D Hamaide J: The power of using functional fMRI on small rodents to study brain pharmacology and disease. *Front Pharmacol.* 2015; 6: 231. 10.3389/fphar.2015.00231 26539115 PMC4612660

[39] Jonckers E van der Linden A Verhoye M: Functional magnetic resonance imaging in rodents: An unique tool to study *in vivo* pharmacologic neuromodulation. *Curr Opin Pharmacol.* 2013; 13(5): 813–20. 10.1016/j.coph.2013.06.008 23856429

[40] Nasseef MT Singh JP Ehrlich AT: Oxycodone-Mediated Activation of the Mu Opioid Receptor Reduces Whole Brain Functional Connectivity in Mice. *ACS Pharmacol Transl Sci.* 2019; 2(4): 264–74. 10.1021/acsptsci.9b00021 32259060 PMC7088903

[41] Keith DE Anton B Murray SR: μ-Opioid Receptor Internalization: Opiate Drugs Have Differential Effects on a Conserved Endocytic Mechanism *In Vitro* and in the Mammalian Brain. *Mol Pharmacol.* 1998; 53(3): 377–84. 10.1124/mol.53.3.377 9495801

[42] Sternini C Spann M Anton B: Agonist-selective endocytosis of mu opioid receptor by neurons in vivo. *Proc Natl Acad Sci U S A.* 1996; 93(17): 9241–6. 10.1073/pnas.93.17.9241 8799185 PMC38626

[43] Kenakin T: Measurement of Receptor Signaling Bias. *Curr Protoc Pharmaco.* 2016; 74: 2.15.1–2.15.15. 10.1002/cpph.11 27636109

[44] Smith JS Lefkowitz RJ Rajagopal S: Biased signalling: From simple switches to allosteric microprocessors. *Nat Rev Drug Discov.* 2018; 17(4): 243–60. 10.1038/nrd.2017.229 29302067 PMC5936084

[45] Ehrlich AT Semache M Gross F: Biased Signaling of the Mu Opioid Receptor Revealed in Native Neurons. *iScience.* 2019; 14: 47–57. 10.1016/j.isci.2019.03.011 30925410 PMC6439305

[46] Whistler JL von Zastrow M: Morphine-activated opioid receptors elude desensitization by beta-arrestin. *Proc Natl Acad Sci U S A.* 1998; 95(17): 9914–9. 10.1073/pnas.95.17.9914 9707575 PMC21436

[47] Leff ER Arttamangkul S Williams JT: Chronic Treatment with Morphine Disrupts Acute Kinase-Dependent Desensitization of GPCRs. *Mol Pharmacol.* 2020; 98(4): 497–507. 10.1124/mol.119.119362 32362586 PMC7562982

[48] Birdsong WT Arttamangkul S Bunzow JR: Agonist Binding and Desensitization of the μ-Opioid Receptor Is Modulated by Phosphorylation of the C-Terminal Tail Domain. *Mol Pharmacol.* 2015; 88(4): 816–24. 10.1124/mol.114.097527 25934731 PMC4576685

[49] Miess E Gondin AB Yousuf A: Multisite phosphorylation is required for sustained interaction with GRKs and arrestins during rapid μ-opioid receptor desensitization. *Sci Signal.* 2018; 11(539): eaas9609. 10.1126/scisignal.aas9609 30018083

[50] Raehal KM Schmid CL Groer CE: Functional selectivity at the μ-opioid receptor: Implications for understanding opioid analgesia and tolerance. *Pharmacol Rev.* 2011; 63(4): 1001–19. 10.1124/pr.111.004598 21873412 PMC3186080

[51] Lohse MJ Benovic JL Codina J: beta-Arrestin: A protein that regulates beta-adrenergic receptor function. *Science.* 1990; 248(4962): 1547–50. 10.1126/science.2163110 2163110

[52] Eichel K von Zastrow M: Subcellular Organization of GPCR Signaling. *Trends Pharmacol Sci.* 2018; 39(2): 200–8. 10.1016/j.tips.2017.11.009 29478570 PMC5830169

[53] Bohn LM Lefkowitz RJ Gainetdinov RR: Enhanced morphine analgesia in mice lacking beta-arrestin 2. *Science.* 1999; 286(5449): 2495–8. 10.1126/science.286.5449.2495 10617462

[54] Gardon O Faget L Chu Sin Chung P: Expression of mu opioid receptor in dorsal diencephalic conduction system: New insights for the medial habenula. *Neuroscience.* 2014; 277: 595–609. 10.1016/j.neuroscience.2014.07.053 25086313 PMC4164589

[55] Erbs E Faget L Scherrer G: A mu-delta opioid receptor brain atlas reveals neuronal co-occurrence in subcortical networks. *Brain Struct Funct.* 2015; 220(2): 677–702. 10.1007/s00429-014-0717-9 24623156 PMC4341027

[56] Märtin A Calvigioni D Tzortzi O: A Spatiomolecular Map of the Striatum. *Cell Rep.* 2019; 29(13): 4320–4333.e5. 10.1016/j.celrep.2019.11.096 31875543

[57] Okunomiya T Hioki H Nishimura C: Generation of a MOR-CreER knock-in mouse line to study cells and neural circuits involved in mu opioid receptor signaling. *Genesis.* 2020; 58(1): e23341. 10.1002/dvg.23341 31651080

[58] Bailly J Del Rossi N Runtz L: Targeting Morphine-Responsive Neurons: Generation of a Knock-In Mouse Line Expressing Cre Recombinase from the Mu-Opioid Receptor Gene Locus. *eNeuro.* 2020; 7(3): ENEURO.0433-19.2020. 10.1523/ENEURO.0433-19.2020 32381649 PMC7266138

[59] Chen XT Pitis P Liu G: Structure-activity relationships and discovery of a G protein biased μ opioid receptor ligand, (3-methoxythiophen-2-yl)methyl({2-(9*R*)-9-(pyridin-2-yl)-6-oxaspiro-4.5decan-9-ylethyl})amine (TRV130), for the treatment of acute severe pain. *J Med Chem.* 2013; 56(20): 8019–31. 10.1021/jm4010829 24063433

[60] Manglik A Lin H Aryal DK: Structure-based discovery of opioid analgesics with reduced side effects. *Nature.* 2016; 537(7619): 185–90. 10.1038/nature19112 27533032 PMC5161585

[61] Schmid CL Kennedy NM Ross NC: Bias Factor and Therapeutic Window Correlate to Predict Safer Opioid Analgesics. *Cell.* 2017; 171(5): 1165–1175.e13. 10.1016/j.cell.2017.10.035 29149605 PMC5731250

[62] Hill R Disney A Conibear A: The novel μ-opioid receptor agonist PZM21 depresses respiration and induces tolerance to antinociception. *Br J Pharmacol.* 2018; 175(13): 2653–61. 10.1111/bph.14224 29582414 PMC6003631

[63] Gillis A Gondin AB Kliewer A: Low intrinsic efficacy for G protein activation can explain the improved side effect profiles of new opioid agonists. *Sci Signal.* 2020; 13(625): eaaz3140. 10.1126/scisignal.aaz3140 32234959

[64] Araldi D Ferrari LF Levine JD: Mu-opioid Receptor (MOR) Biased Agonists Induce Biphasic Dose-dependent Hyperalgesia and Analgesia, and Hyperalgesic Priming in the Rat. *Neuroscience.* 2018; 394: 60–71. 10.1016/j.neuroscience.2018.10.015 30342200 PMC6261666

[65] Altarifi AA David B Muchhala KH: Effects of acute and repeated treatment with the biased mu opioid receptor agonist TRV130 (oliceridine) on measures of antinociception, gastrointestinal function, and abuse liability in rodents. *J Psychopharmacol.* 2017; 31(6): 730–9. 10.1177/0269881116689257 28142305 PMC5646680

[66] Austin Zamarripa C Edwards SR Qureshi HN: The G-protein biased mu-opioid agonist, TRV130, produces reinforcing and antinociceptive effects that are comparable to oxycodone in rats. *Drug Alcohol Depend.* 2018; 192: 158–62. 10.1016/j.drugalcdep.2018.08.002 30261403 PMC6223023

[67] Montandon G Ren J Victoria NC: G-protein-gated Inwardly Rectifying Potassium Channels Modulate Respiratory Depression by Opioids. *Anesthesiology.* 2016; 124(3): 641–50. 10.1097/ALN.0000000000000984 26675532 PMC4755838

[68] Conibear AE Kelly E: A Biased View of *μ*-Opioid Receptors? *Mol Pharmacol.* 2019; 96(5): 542–9. 10.1124/mol.119.115956 31175184 PMC6784500

[69] Kliewer A Schmiedel F Sianati S: Phosphorylation-deficient G-protein-biased μ-opioid receptors improve analgesia and diminish tolerance but worsen opioid side effects. *Nat Commun.* 2019; 10(1): 367. 10.1038/s41467-018-08162-1 30664663 PMC6341117

[70] Kliewer A Gillis A Hill R: Morphine-induced respiratory depression is independent of β-arrestin2 signalling. *Br J Pharmacol.* 2020; 177(13): 2923–31. 10.1111/bph.15004 32052419 PMC7280004

[71] Singla NK Skobieranda F Soergel DG: APOLLO-2: A Randomized, Placebo and Active-Controlled Phase III Study Investigating Oliceridine (TRV130), a G Protein-Biased Ligand at the μ-Opioid Receptor, for Management of Moderate to Severe Acute Pain Following Abdominoplasty. *Pain Pract.* 2019; 19(7): 715–31. 10.1111/papr.12801 31162798 PMC6851842

[72] Gan TJ Wase L: Oliceridine, a G protein-selective ligand at the μ-opioid receptor, for the management of moderate to severe acute pain. *Drugs Today (Barc).* 2020; 56(4): 269–86. 10.1358/dot.2020.56.4.3107707 32309822

[73] Ayad S Demitrack MA Burt DA: Evaluating the Incidence of Opioid-Induced Respiratory Depression Associated with Oliceridine and Morphine as Measured by the Frequency and Average Cumulative Duration of Dosing Interruption in Patients Treated for Acute Postoperative Pain. *Clin Drug Investig.* 2020; 40(8): 755–64. 10.1007/s40261-020-00936-0 32583295 PMC7359152

[74] Black JW Leff P: Operational models of pharmacological agonism. *Proc R Soc Lond B Biol Sci.* 1983; 220(1219): 141–62. 10.1098/rspb.1983.0093 6141562

[75] McPherson J Rivero G Baptist M: μ-opioid receptors: Correlation of agonist efficacy for signalling with ability to activate internalization. *Mol Pharmacol.* 2010; 78(4): 756–66. 10.1124/mol.110.066613 20647394 PMC2981392

[76] Stahl EL Bohn LM: Re-evaluating how low intrinsic efficacy and apparent bias for G protein activation relates to the improved side effect profiles of new opioid agonists. *bioRxiv.* 2020; 2020.11.19.390518. 10.1101/2020.11.19.390518

[77] Gomes I Sierra S Lueptow L: Biased signaling by endogenous opioid peptides. *Proc Natl Acad Sci U S A.* 2020; 117(21): 11820–8. 10.1073/pnas.2000712117 32393639 PMC7261131

[78] Ehrlich AT Darcq E: Recommending buprenorphine for pain management. *Pain Manag.* 2019; 9(1): 13–6. 10.2217/pmt-2018-0069 30507294

[79] Johnson RE Fudala PJ Payne R: Buprenorphine: Considerations for pain management. *J Pain Symptom Manage.* 2005; 29(3): 297–326. 10.1016/j.jpainsymman.2004.07.005 15781180

[80] Kyzer JL Wenthur CJ: Classics in Chemical Neuroscience: Buprenorphine. *ACS Chem Neurosci.* 2020; 11(10): 1385–99. 10.1021/acschemneuro.0c00100 32302475

[81] Aiyer R Gulati A Gungor S: Treatment of Chronic Pain With Various Buprenorphine Formulations: A Systematic Review of Clinical Studies. *Anesth Analg.* 2018; 127(2): 529–38. 10.1213/ANE.0000000000002718 29239947

[82] Scherrer G Tryoen-Tóth P Filliol D: Knockin mice expressing fluorescent delta-opioid receptors uncover G protein-coupled receptor dynamics *in vivo*. *Proc Natl Acad Sci U S A.* 2006; 103(25): 9691–6. 10.1073/pnas.0603359103 16766653 PMC1480468

[83] Maduna T Audouard E Dembélé D: Microglia Express Mu Opioid Receptor: Insights From Transcriptomics and Fluorescent Reporter Mice. *Front Psychiatry.* 2018; 9: 726. 10.3389/fpsyt.2018.00726 30662412 PMC6328486

[84] Nam MH Han KS Lee J: Expression of µ-Opioid Receptor in CA1 Hippocampal Astrocytes. *Exp Neurobiol.* 2018; 27(2): 120–8. 10.5607/en.2018.27.2.120 29731678 PMC5934543

[85] Corder G Tawfik VL Wang D: Loss of μ opioid receptor signaling in nociceptors, but not microglia, abrogates morphine tolerance without disrupting analgesia. *Nat Med.* 2017; 23(2): 164–73. 10.1038/nm.4262 28092666 PMC5296291

[86] Darcq E Koebel P Del Boca C: RSK2 signaling in brain habenula contributes to place aversion learning. *Learn Mem.* 2011; 18(9): 574–8. 10.1101/lm.2221011 21852432 PMC3256568

[87] Kobayashi Y Sano Y Vannoni E: Genetic dissection of medial habenula-interpeduncular nucleus pathway function in mice. *Front Behav Neurosci.* 2013; 7: 17. 10.3389/fnbeh.2013.00017 23487260 PMC3594921

[88] Otsu Y Darcq E Pietrajtis K: Control of aversion by glycine-gated GluN1/GluN3A NMDA receptors in the adult medial habenula. *Science.* 2019; 366(6462): 250–4. 10.1126/science.aax1522 31601771 PMC7556698

[89] Boulos LJ Ben Hamida S Bailly J: Mu opioid receptors in the medial habenula contribute to naloxone aversion. *Neuropsychopharmacology.* 2020; 45(2): 247–55. 10.1038/s41386-019-0395-7 31005059 PMC6901535

[90] Darcq E Befort K Koebel P: RSK2 signaling in medial habenula contributes to acute morphine analgesia. *Neuropsychopharmacology.* 2012; 37(5): 1288–96. 10.1038/npp.2011.316 22218090 PMC3306890

[91] Ugur M Derouiche L Massotte D: Heteromerization Modulates mu Opioid Receptor Functional Properties *in vivo*. *Front Pharmacol.* 2018; 9: 1240. 10.3389/fphar.2018.01240 30483121 PMC6244869

[92] Fujita W Gomes I Devi LA: Heteromers of μ-δ opioid receptors: New pharmacology and novel therapeutic possibilities. *Br J Pharmacol.* 2015; 172(2): 375–87. 10.1111/bph.12663 24571499 PMC4292954

[93] Wang D Tawfik VL Corder G: Functional Divergence of Delta and Mu Opioid Receptor Organization in CNS Pain Circuits. *Neuron.* 2018; 98(1): 90–108.e5. 10.1016/j.neuron.2018.03.002 29576387 PMC5896237

[94] Degrandmaison J Abdallah K Blais V: *in vivo* mapping of a GPCR interactome using knockin mice. *Proc Natl Acad Sci U S A.* 2020; 117(23): 13105–16. 10.1073/pnas.1917906117 32457152 PMC7293596

[95] Huang W Manglik A Venkatakrishnan AJ: Structural insights into µ-opioid receptor activation. *Nature.* 2015; 524(7565): 315–21. 10.1038/nature14886 26245379 PMC4639397

[96] Koehl A Hu H Maeda S: Structure of the µ-opioid receptor-Gi protein complex. *Nature.* 2018; 558(7711): 547–52. 10.1038/s41586-018-0219-7 29899455 PMC6317904

[97] Nehmé R Carpenter B Singhal A: Mini-G proteins: Novel tools for studying GPCRs in their active conformation. *PLoS One.* 2017; 12(4): e0175642. 10.1371/journal.pone.0175642 28426733 PMC5398546

[98] Wan Q Okashah N Inoue A: Mini G protein probes for active G protein-coupled receptors (GPCRs) in live cells. *J Biol Chem.* 2018; 293(19): 7466–73. 10.1074/jbc.RA118.001975 29523687 PMC5949987

[99] Stoeber M Jullié D Lobingier BT: A Genetically Encoded Biosensor Reveals Location Bias of Opioid Drug Action. *Neuron.* 2018; 98(5): 963–976.e5. 10.1016/j.neuron.2018.04.021 29754753 PMC6481295

[100] Stoeber M Jullié D Li J: Agonist-selective recruitment of engineered protein probes and of GRK2 by opioid receptors in living cells. *Elife.* 2020; 9: e54208. 10.7554/eLife.54208 32096468 PMC7041944

[101] Váradi A Marrone GF Palmer TC: Mitragynine/Corynantheidine Pseudoindoxyls As Opioid Analgesics with Mu Agonism and Delta Antagonism, Which Do Not Recruit β-Arrestin-2. *J Med Chem.* 2016; 59(18): 8381–97. 10.1021/acs.jmedchem.6b00748 27556704 PMC5344672

[102] Arttamangkul S Plazek A Platt EJ: Visualizing endogenous opioid receptors in living neurons using ligand-directed chemistry. *Elife.* 2019; 8: e49319. 10.7554/eLife.49319 31589142 PMC6809603

[103] Thomsen ARB Plouffe B Cahill TJ: GPCR-G Protein-β-Arrestin Super-Complex Mediates Sustained G Protein Signaling. *Cell.* 2016; 166(4): 907–19. 10.1016/j.cell.2016.07.004 27499021 PMC5418658

[104] Irannejad R Tomshine JC Tomshine JR: Conformational biosensors reveal GPCR signalling from endosomes. *Nature.* 2013; 495(7442): 534–8. 10.1038/nature12000 23515162 PMC3835555

[105] Tsvetanova NG von Zastrow M: Spatial encoding of cyclic AMP signaling specificity by GPCR endocytosis. *Nat Chem Biol.* 2014; 10(12): 1061–5. 10.1038/nchembio.1665 25362359 PMC4232470

[106] Calebiro D Nikolaev VO Gagliani MC: Persistent cAMP-signals triggered by internalized G-protein-coupled receptors. *PLoS Biol.* 2009; 7(8): e1000172. 10.1371/journal.pbio.1000172 19688034 PMC2718703

[107] Aldrich JV McLaughlin JP: Opioid peptides: Potential for drug development. *Drug Discov Today Technol.* 2012; 9(1): e23–e31. 10.1016/j.ddtec.2011.07.007 23316256 PMC3539827

[108] de Marco R Janecka A: Strategies to Improve Bioavailability and *In Vivo* Efficacy of the Endogenous Opioid Peptides Endomorphin-1 and Endomorphin-2. *Curr Top Med Chem.* 2015; 16(2): 141–55. 10.2174/1568026615666150817103635 26279081

[109] Olson KM Lei W Keresztes A: Novel Molecular Strategies and Targets for Opioid Drug Discovery for the Treatment of Chronic Pain. *Yale J Biol Med.* 2017; 90(1): 97–110. 28356897 PMC5369049

[110] Jullié D Stoeber M Sibarita JB: A Discrete Presynaptic Vesicle Cycle for Neuromodulator Receptors. *Neuron.* 2020; 105(4): 663–677.e8. 10.1016/j.neuron.2019.11.016 31837915 PMC7035187

[111] Paek J Kalocsay M Staus DP: Multidimensional Tracking of GPCR Signaling via Peroxidase-Catalyzed Proximity Labeling. *Cell.* 2017; 169(2): 338–349.e11. 10.1016/j.cell.2017.03.028 28388415 PMC5514552

[112] Lobingier BT Hüttenhain R Eichel K: An Approach to Spatiotemporally Resolve Protein Interaction Networks in Living Cells. *Cell.* 2017; 169(2): 350–360.e12. 10.1016/j.cell.2017.03.022 28388416 PMC5616215

[113] Jimenez-Vargas NN Gong J Wisdom MJ: Endosomal signaling of delta opioid receptors is an endogenous mechanism and therapeutic target for relief from inflammatory pain. *Proc Natl Acad Sci U S A.* 2020; 117(26): 15281–92. 10.1073/pnas.2000500117 32546520 PMC7334524

[114] Atasoy D Aponte Y Su HH: A FLEX switch targets Channelrhodopsin-2 to multiple cell types for imaging and long-range circuit mapping. *J Neurosci.* 2008; 28(28): 7025–30. 10.1523/JNEUROSCI.1954-08.2008 18614669 PMC2593125

[115] Tye KM Deisseroth K: Optogenetic investigation of neural circuits underlying brain disease in animal models. *Nat Rev Neurosci.* 2012; 13(4): 251–66. 10.1038/nrn3171 22430017 PMC6682316

[116] Roth BL: DREADDs for Neuroscientists. *Neuron.* 2016; 89(4): 683–94. 10.1016/j.neuron.2016.01.040 26889809 PMC4759656

[117] Seshadri S Hoeppner DJ Tajinda K: Calcium Imaging in Drug Discovery for Psychiatric Disorders. *Front Psychiatry.* 2020; 11: 713. 10.3389/fpsyt.2020.00713 32793004 PMC7390878

[118] Bruchas MR Roth BL: New Technologies for Elucidating Opioid Receptor Function. *Trends Pharmacol Sci.* 2016; 37(4): 279–89. 10.1016/j.tips.2016.01.001 26833118 PMC4811714

[119] Al-Hasani R McCall JG Shin G: Distinct Subpopulations of Nucleus Accumbens Dynorphin Neurons Drive Aversion and Reward. *Neuron.* 2015; 87(5): 1063–77. 10.1016/j.neuron.2015.08.019 26335648 PMC4625385

[120] Harris JA Hirokawa KE Sorensen SA: Anatomical characterization of Cre driver mice for neural circuit mapping and manipulation. *Front Neural Circuits.* 2014; 8: 76. 10.3389/fncir.2014.00076 25071457 PMC4091307

[121] Cai X Huang H Kuzirian MS: Generation of a *KOR-Cre* knockin mouse strain to study cells involved in kappa opioid signaling. *Genesis.* 2016; 54(1): 29–37. 10.1002/dvg.22910 26575788 PMC4747253

[122] Bachmutsky I Wei XP Kish E: Opioids depress breathing through two small brainstem sites. *eLife.* 2020; 9: e52694. 10.7554/eLife.52694 32073401 PMC7077984

[123] Corder G Ahanonu B Grewe BF: An amygdalar neural ensemble that encodes the unpleasantness of pain. *Science.* 2019; 363(6424): 276–81. 10.1126/science.aap8586 30655440 PMC6450685

[124] Chuang KH Nasrallah FA: Functional networks and network perturbations in rodents. *Neuroimage.* 2017; 163: 419–36. 10.1016/j.neuroimage.2017.09.038 28942060

[125] Bruns A Künnecke B Risterucci C: Validation of cerebral blood perfusion imaging as a modality for quantitative pharmacological MRI in rats. *Magn Reson Med.* 2009; 61(6): 1451–8. 10.1002/mrm.21779 19358231

[126] Bruns A Mueggler T Künnecke B: "Domain gauges": A reference system for multivariate profiling of brain fMRI activation patterns induced by psychoactive drugs in rats. *Neuroimage.* 2015; 112: 70–85. 10.1016/j.neuroimage.2015.02.032 25724758

[127] Ferrari L Turrini G Crestan V: A robust experimental protocol for pharmacological fMRI in rats and mice. *J Neurosci Methods.* 2012; 204(1): 9–18. 10.1016/j.jneumeth.2011.10.020 22068031

[128] Lindemann L Porter RH Scharf SH: Pharmacology of basimglurant (RO4917523, RG7090), a unique metabotropic glutamate receptor 5 negative allosteric modulator in clinical development for depression. *J Pharmacol Exp Ther.* 2015; 353(1): 213–33. 10.1124/jpet.114.222463 25665805

[129] Becerra L Harter K Gonzalez RG: Functional magnetic resonance imaging measures of the effects of morphine on central nervous system circuitry in opioid-naive healthy volunteers. *Anesth Analg.* 2006; 103(1): 208–16. table of contents. 10.1213/01.ane.0000221457.71536.e0 16790655

[130] Gorka SM Fitzgerald DA de Wit H: Opioid modulation of resting-state anterior cingulate cortex functional connectivity. *J Psychopharmacol.* 2014; 28(12): 1115–24. 10.1177/0269881114548436 25237122 PMC5613932

[131] Mechling AE Arefin T Lee HL: Deletion of the mu opioid receptor gene in mice reshapes the reward-aversion connectome. *Proc Natl Acad Sci U S A.* 2016; 113(41): 11603–8. 10.1073/pnas.1601640113 27671662 PMC5068324

[132] Moore K Madularu D Iriah S: BOLD Imaging in Awake Wild-Type and Mu-Opioid Receptor Knock-Out Mice Reveals On-Target Activation Maps in Response to Oxycodone. *Front Neurosci.* 2016; 10: 471. 10.3389/fnins.2016.00471 27857679 PMC5094148

[133] Mandino F Cerri DH Garin CM: Animal Functional Magnetic Resonance Imaging: Trends and Path Toward Standardization. *Front Neuroinform.* 2019; 13: 78. 10.3389/fninf.2019.00078 32038217 PMC6987455

[134] Zhou W Jin Y Meng Q: A neural circuit for comorbid depressive symptoms in chronic pain. *Nat Neurosci.* 2019; 22(10): 1649–58. 10.1038/s41593-019-0468-2 31451801

[135] Grandjean J Corcoba A Kahn MC: A brain-wide functional map of the serotonergic responses to acute stress and fluoxetine. *Nat Commun.* 2019; 10(1): 350. 10.1038/s41467-018-08256-w 30664643 PMC6341094

[136] Peeters LM Hinz R Detrez JR: Chemogenetic silencing of neurons in the mouse anterior cingulate area modulates neuronal activity and functional connectivity. *Neuroimage.* 2020; 220: 117088. 10.1016/j.neuroimage.2020.117088 32592851

[137] Benekareddy M Stachniak TJ Bruns A: Identification of a Corticohabenular Circuit Regulating Socially Directed Behavior. *Biol Psychiatry.* 2018; 83(7): 607–17. 10.1016/j.biopsych.2017.10.032 29336819

[138] Giorgi A Migliarini S Galbusera A: Brain-wide Mapping of Endogenous Serotonergic Transmission via Chemogenetic fMRI. *Cell Rep.* 2017; 21(4): 910–8. 10.1016/j.celrep.2017.09.087 29069598

